# Single-Incision Laparoscopy Surgery Excision of an Infected Urachal Cyst: Description of the Technique

**DOI:** 10.1089/cren.2016.0129

**Published:** 2017-01-01

**Authors:** Juan D. Garisto, Katherine Henriquez, Edwin E. Pimentel M.

**Affiliations:** Department of Urology, Complejo Hospitalario Metropolitano Dr. Arnulfo Arias Madrid, Panama City, Panama.

**Keywords:** urachal cyst, minimal invasive, infection

## Abstract

***Background:*** Urachal cysts (UCs) are secondary to incomplete obliteration of the embryonic urachal duct and may become symptomatic when infected. Treatment is primarily surgical to excise the infected cyst. Surgical approaches include a lower midline laparotomy or minimally invasive (MI) techniques.

***Case:*** We present a case of a young male with an infected UC that was treated with a single-incision laparoscopy surgery. The operative technique is described.

***Conclusion:*** This approach is a safe and feasible option for the MI management of UCs.

## Introduction

The urachus is the embryologic remnant of the allantois and provides ligamentous support from the dome of the bladder to the umbilicus. Urachal remnants are rare anomalies in adults and can present as an urachal cyst (UC). Infection is a usual mode of appearance developing nonspecific symptoms in young adults such as erythema, lower abdominal pain, fever, dysuria, and purulent umbilical discharge. UC treatments include antibiotic therapy, drainage, and surgical excision of the cyst. Multiple approaches include removal by open, laparoscopic, or robotic surgery. Likewise, the laparoendoscopic single-site (LESS) surgery or single incision laparoscopic surgery (SILS) represents a less invasive access and had been developed to benefit patients by enabling surgeons to perform aesthetic surgery. In the present case, we describe the technique of the LESS surgery used to manage an infected UC in a young male.

## Case Presentation

**FIG. 1.**
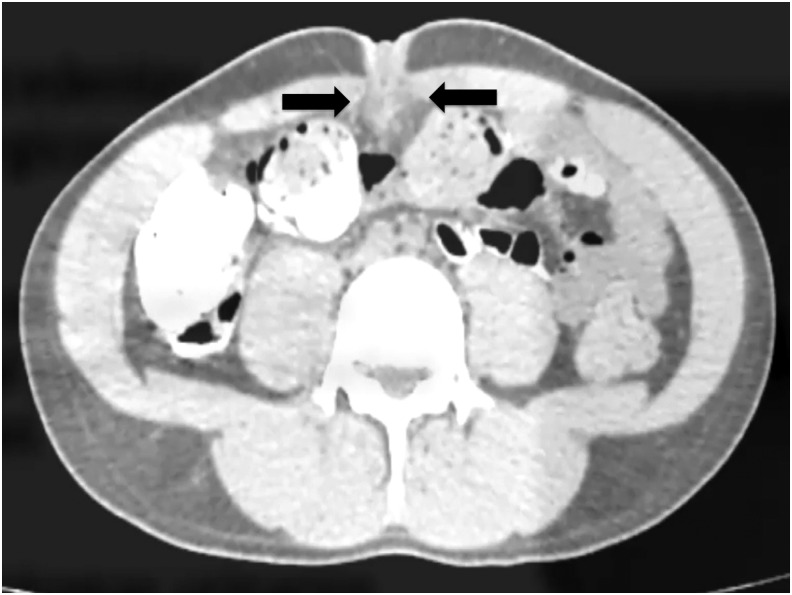
Abdominopelvic CT scan shows a 2.5 × 2.5 midline cystic mass (*arrows*) with inflammatory changes.

A 25-year-old Hispanic man presented to the emergency department with 3 weeks mild abdominal pain and purulent discharge from the umbilicus. He denied any urinary tract symptoms and medical history. Physical examination revealed periumbilical redness with umbilical discharge. Upon admission to the hospital, the patient had a body temperature of 38.6°C, blood pressure of 120/70 mm Hg, heart rate of 90 beats/minute, and respiratory rate of 14 breaths/minute. Laboratory results included hemoglobin, 12.5 g/dL; white blood cell count, 13,400/mm^3^; and platelets, 255,000/mm^3^. Abdominopelvic CT detected an infraumbilical cystic mass in the midline with inflammatory changes extending to the bladder dome (Fig. 1). Treatment with antibiotics was started before elective surgery.

## Surgical Technique

**FIG. 2.**
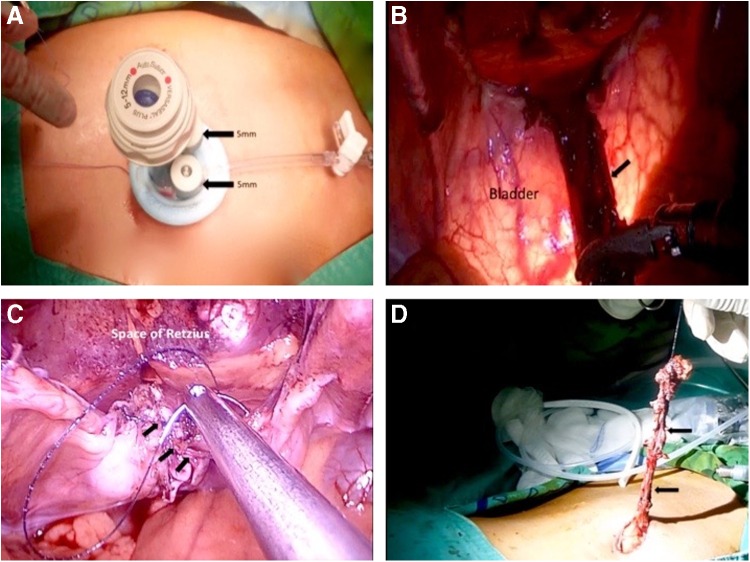
**(A)** Transumbilical SILS™ port was introduced into the abdominal cavity. Three trocars were inserted, two for 5 mm ports and one for a 12 mm port; **(B)**UC excision using a Double-approach. Notice the transilumination of bladder assisted with a flexible cystoscope; **(C)** Watertight continuous closure (*arrows*) of the bladder dome using V-Lock; **(D)** Urachus and bladder cuff (*arrows*) were removed through the surgical wound site. UC, urachal cyst.

**FIG. 3.**
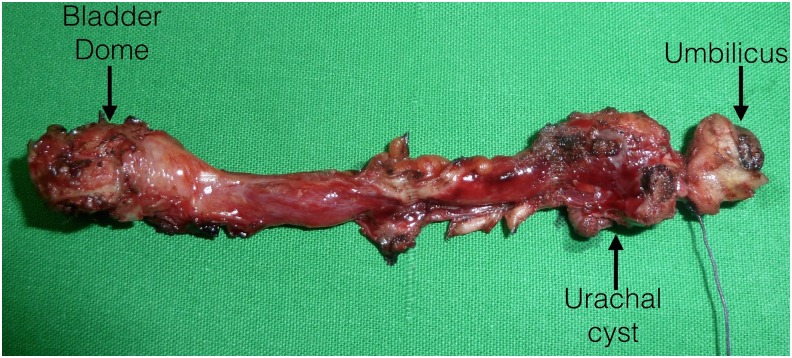
Infected UC.

The patient was positioned supine on the operating table with his legs spread in a 30° Trendelenburg position. An 18F Foley catheter was inserted to empty the bladder. We conducted a curvilinear incision surrounding the umbilical fold. The umbilical ligament was identified under direct vision and transected at the base of the umbilicus. The dissected 3 cm of the proximal urachal remnant was dropped intraperitoneally, hanging down from the anterior abdominal wall. By an open technique, a transperitoneal SILS™ port (Covidien, Norwalk, CT) was introduced to the abdominal cavity through the incision. Through this multiport, two 5 mm trocars and a 12 mm 30° lens rigid camera were inserted (Fig. 2A). Pneumoperitoneum was induced with carbon dioxide infusion to a pressure of 12 mm Hg. Preperitoneal urachal remnant was resected distally to the roof of the bladder using Roticulator™ (Covidien, Norwalk, CT). To guide the circumferential resection of the bladder dome (Fig. 2B), a cystoscope was inserted into the bladder. Partial cystectomy was followed by a bladder repair watertight with continuous 3-0 V-Lock one layer running suture (Fig. 2C). Urachal remnant and bladder cuff were removed with the umbilicus (Fig. 2D). Jackson–Pratt drainage was placed on the surgical field and the periumbilical skin was attached to rectus fascia with absorbable buried sutures to make a neoumbilicus. There were no operative complications. Estimated blood loss was minimal, and the intraoperative time was 180 minutes. The postoperative course was uneventful and he was discharged on the first day after operation. Bladder catheter was removed 7 days after cystogram. At 6 months follow-up, the patient remained well with normal activity and good cosmetic results. Pathology report determined the specimen as an infected benign UC (Fig. 3).

## Discussion

Urachal anomalies are present in as much as 2% of the general population. They are secondary to incomplete obliteration of the urachus during embryonic development. Moreover, these abnormalities are found twice as frequently in males and rarely presents in adults at ages 20–40 years. The persistency of the urachus generates a number of configurations depending on the location and degree of obliteration such as UC (39%), urachus patent (29%), urachal sinus (22%), and vesicourachal diverticulum (6%). Of these, UCs are most often detected as a result of an infection with periumbilical inflammation. Also, they can develop an abscess with drainage either through the umbilicus or bladder and the potential malignant degeneration.

Traditionally, CT and ultrasonography are ideally image modalities capable of establishing UCs because they display helpful cross-sectional images in the anterior abdominal wall away from interfering bowel structures. Cystoscopy is useful to determine a patent bladder communication and take biopsies if necessary.

The conventional treatment for the infected UC combines initial antibiotic treatment follows by drainage of the cyst or the purulent collections. Afterward, a radical preperitoneal excision of the urachal remnant needs to be achieved to prevent the possibility of complications, including cyst recurrence or malignant degeneration. The use of flexible laparoscopic instruments and camera in LESS surgery depends on the preference of the surgeon. The Roticulator can be angled at the tip to allow triangulation during the dissection of the urachus. We recommend the use of a light projected from the cystoscopy to identify the urachus insertion, to guide the resection of the bladder cuff, and to ensure a safe distal closure.

Different surgical access for removal of UC had been described. Historically, an open procedure using a lower midline incision illustrates a safe and efficient approach. However, minimally invasive (MI) techniques have gained increasing acceptance for child and young adult patients. Trondsen described the first case with standard laparoscopy in 1993, and Patrzyk^1^ with LESS surgery in 2010. Description of the laparoscopic management for the urachal abnormality can be done using three ports. Multiple reports indicate a 12-mm camera port placed in the midline above the umbilicus and a 5 and 12-mm working ports placed on the lateral border of the rectus muscle. An alternative port placement consists in all three ports inserted in a horizontal line of the left lateral margin of the rectus muscle.

In addition, robot-assisted surgery for resection of the UC had also been reported. Advantages comparing the laparoscopic and open surgery include the ergonomics, three-dimensional observation, and simplicity of suturing the bladder. Three incisions are needed to gain access previous to docking the robot: two 8-mm robotic arms ports are inserted at the same level of the umbilicus and at the lateral margin of the rectus muscle and a 12-mm camera port is inserted 5 cm above the umbilicus. Both techniques, robotic and “pure laparoscopic,” provide a favorable alternative for the management of an infected urachus.

LESS surgery potentially reduces morbidity and scarring associated with other approaches. It is more complex and challenging than the standard laparoscopic technique because of the proximity of each trocar and the use in mirror of flexible instruments.^2^ SILS may be an option in select patients with urachal anomalies in hospitals without the assistance of a robot.

Our report details a particular variant of the conventional MI techniques. Rather than the traditional three small incisions, SILS employs a single and unique small incision at the entry point, providing the benefits of fewer scars, less pain, and shorter recovery periods.^3^ It also represents a less expensive procedure compared with the costs of the robotic approach and less invasiveness with better cosmetic result than the pure laparoscopic approach.

## Conclusion

We described the techniques used for single-incision laparoscopy surgery of an infected UC and bladder cuff with bladder repair. This approach proved to be a safe and effective and satisfactory cosmetic option for the MI management of UCs in hospitals without a robot.

## Abbreviations Used

CT: computed tomography
LESS: laparoendoscopic single-site
MI: minimally invasive
SILS: single incision laparoscopic surgery
UC: urachal cyst
